# Expanding the Clinical and Mutational Spectrum of *FBXO7*-Related Parkinsonism: A Novel Italian Family and Comprehensive Literature Review

**DOI:** 10.3390/genes17070764

**Published:** 2026-06-30

**Authors:** Stefania Zampatti, Claudia Strafella, Rosa Campopiano, Cristina Peconi, Juliette Farro, Francesca Chiara De Pinto, Roberta Fantozzi, Nicola Modugno, Stefano Gambardella, Carlo Caltagirone, Emiliano Giardina

**Affiliations:** 1 Genomic Medicine Laboratory, IRCCS Fondazione Santa Lucia, 00179 Rome, RM, Italy; 2 IRCCS Neuromed, 86077 Pozzilli, IS, Italy; 3 Department of Biomolecular Sciences, University of Urbino, 61029 Urbino, PU, Italy; 4 Department of Clinical and Behavioral Neurology, IRCCS Fondazione Santa Lucia, 00179 Rome, RM, Italy; 5 Department of Biomedicine and Prevention, Tor Vergata University, 00133 Rome, RM, Italy

**Keywords:** PARK15, Parkinsonian-Pyramidal Syndrome, early-onset Parkinson disease

## Abstract

Background: Mutations in the *FBXO7* gene (PARK15) cause an autosomal recessive, early-onset neurodegenerative disorder typically presenting as Parkinsonian-Pyramidal Syndrome (PPS). Despite its recognition, the high phenotypic variability often delays diagnosis. Here, we report a novel Italian family and synthesize data from all published cases to date, offering an updated clinical and molecular overview of the disease. Methods: We performed clinical and molecular characterization of a newly identified family. Furthermore, we conducted a systematic literature review (from 2008 to 2026) to aggregate clinical, genetic, and geographic data of all reported PARK15 cases. Results: Two siblings presented with a complex phenotype including early-onset parkinsonism, cognitive decline, psychiatric symptoms, and aphasia-type speech disorders. Genetic analyses identified two novel likely pathogenic variants: a missense substitution in the UBL domain (p.Ile74Met) and a frameshift indel (p.Val233GlufsTer8). The literature review (incorporating clinical data from Europe, Asia, and South America) confirms a high prevalence of postural instability (87.5%), bradykinesia (83.3%), and pyramidal signs (~60%). We observed a distinct distribution of variants: missense mutations cluster in the N-terminal UBL and F-box domains, while truncating variants are more common in the C-terminal region. Discussion: Our findings expand the *FBXO7* mutational landscape and underscore the “atypical” clinical markers, such as pyramidal signs and cognitive decline, that distinguish PARK15 from other recessive forms of parkinsonism like PARK2 and PARK6. The dual role of FBXO7 in mitochondrial quality control and proteasomal assembly suggests a broad disruption of cellular homeostasis. These observations refine genotype–phenotype correlations and may guide variant interpretation in routine diagnostic settings.

## 1. Introduction

Mutations in the *FBXO7* gene (*F-box only protein 7*, also known as *PARK15*) lead to an autosomal recessive, early-onset neurodegenerative disorder that typically presents with a complex parkinsonian-pyramidal phenotype. Once called Parkinsonian-Pyramidal Syndrome (PPS) or Davison’s syndrome [1,2,3,4], it differs from idiopathic Parkinson’s disease by the presence of both extrapyramidal (parkinsonian) and pyramidal (spasticity-related) signs [5]. *FBXO7*-associated disease, known as *PARK15*, is a distinct clinical entity marked by a range of progressive neurodegenerative symptoms that usually appear in childhood or early adulthood. Its primary clinical feature is the coexistence of extrapyramidal and pyramidal system involvement, resulting in the PPS:

- Parkinsonism: Patients typically develop signs of Parkinson’s disease, including bradykinesia, rigidity, and postural instability. Notably, tremor is often less prominent than in typical PD.
- Pyramidal Signs: These symptoms, resulting from damage to the corticospinal tracts, include spastic paraplegia, hyperreflexia, and the Babinski sign.

Although there is significant phenotypic variability, the disease typically presents in the juvenile or early-adult age group, usually in the second or third decade of life (range: 7 to 52 years). Some families exhibit cognitive decline, supranuclear gaze palsy, and dysarthria. Beyond the main features, patients may exhibit additional neurodegenerative-associated symptoms, including cognitive decline [6,7,8,9,10,11,12,13,14,15,16], ataxia, and nystagmus (which underscore cerebellar degeneration) [14], seizures [10,14,16], brain iron accumulation (NBIA—Neurodegeneration with Brain Iron Accumulation) [14,15,17], psychiatric symptoms (visual hallucinations, aggressive behavior, and impulse control disorders) [7,9,11,13,16,18,19], dystonia, chorea, or perioral myoclonus [7,11,13,16,18,19]. This great phenotypic variability makes diagnosis difficult, and many patients with *FBXO7*-associated disease receive the diagnosis many years after the onset of symptoms [8,11,12].

Understanding the phenotypes associated with pathogenic *FBXO7* variants is critical for early diagnosis and treatment. In fact, Parkinsonism in *FBXO7*-associated disease often shows variable degrees of response to Levodopa therapy, ranging from good initial responsiveness to nonresponsiveness or the development of marked motor fluctuations and Levodopa-induced dyskinesias as the disease progresses. In particular, most patients show an initial favorable response to Levodopa, though this is often complicated by early-onset dyskinesia and psychiatric side effects [8,11,12] (details about response to Levodopa are provided in Supplementary Table S2). The prognosis is often marked by rapidly progressive Parkinsonism and a decline in motor function, leading to significant disability and often wheelchair dependence.

Given the atypical presentation of patients with *FBXO7* pathogenic variants, it is essential to highlight the common and atypical features that could aid differential diagnosis. We report a novel Italian family carrying two previously unreported variants and integrate all available clinical and neuroimaging data, examining the molecular distribution of variants across functional protein domains, geographic patterns, and genotype–phenotype correlations. Moreover, this study aims to provide a comprehensive overview of *FBXO7*-related Parkinsonism by integrating all reported variants and associated clinical and neuroimaging data, focusing on their molecular distribution across functional domains, geographic distribution, and genotype–phenotype correlations.

## 2. Methods

This study combines original clinical and genetic data from a newly identified family with a comprehensive collection of previously reported *FBXO7*-related cases. Newly identified patients were clinically and molecularly characterized, while published data were systematically retrieved and curated. Aggregated data were then integrated to investigate the distribution of variants across functional domains, their geographic occurrence, and genotype–phenotype correlations.

### 2.1. Clinical and Molecular Characterization of a Newly Identified Family

The family was enrolled in routine clinical activity at the IRCCS Neuromed. Neurological and instrumental evaluations were performed for diagnostic purposes. All genetic evaluations were performed after genetic counseling, and all subjects provided written informed consent.

Peripheral venous blood (5 mL) was collected from the index patients, their parents, and the unaffected daughter. Genomic DNA was extracted from whole blood using the QIAamp DNA Blood Mini Kit (QIAGEN GmbH, Hilden, Germany), and nucleic acid quantification was performed using a Qubit 2.0 (Invitrogen, Thermo Fisher Scientific, Waltham, MA, USA). Clinical exome sequencing of 4490 human genes was performed using the Clinical Exome Solution v2 kit (Sophia Genetics, SA, Boston, MA, USA). The resulting libraries were processed for paired-end sequencing on the Illumina NextSeq 550 platform (San Diego, CA, USA). Sophia DDM platform (Sophia Genetics, SA) was used for read alignment on the hg19 reference genome, annotation, prioritization, and selection of potentially pathogenic variants.

Various bioinformatics tools (Sophia DDM platform, enGenome eVai, enGenome VarChat) were employed to predict variant functions and annotate noncoding regulatory sequences. Each variant was classified following the standards and guidelines for sequence variant interpretation published by the American College of Medical Genetics and Genomics. DNA variants were described using HGVS nomenclature. Candidate disease-causing variants were identified by tetra-bioinformatics analysis (two affected siblings and healthy parents) and prioritized based on their consistency with the clinical phenotype and segregation pattern. To further investigate new mutations in *FBXO7*, AlphaFold and DynaMut2 were applied to model the 3D structure of the FBXO7 protein and to obtain a highly accurate 3D model of the wild-type human FBXO7 protein structure, revealing that the mutation I74M falls in the UBL (Ubiquitin-Like) domain. The predicted stability change (ΔΔGStability) was calculated through DynaMut2 on the AF-Q9Y3I1-F1 Model PDB. Protein domain annotation was performed using UniProt and InterPro databases.

Segregation analysis of the whole family (two parents and three siblings, one of whom is healthy) was performed using Sanger sequencing. Flanking regions were amplified using specific primers (Table S1). PCR products were purified by MinElute PCR Purification (QIAGEN GmbH, Hilden, Germany) and sequenced on an SeqStudio Genetic Analyzer (Applied Biosystems by Thermo Fisher Scientific, Waltham, MA, USA) using the BigDye Terminator v3.1 Kit (Thermo Fisher Scientific, Waltham, MA, USA).

### 2.2. Review of Published Cases and Data Extraction

A systematic and comprehensive literature review was conducted to identify all published reports of *FBXO7*/PARK15-related cases in the PubMed database, including both single-case reports and patient cohorts, from the first description [20] to the present. The search string used a combination of Medical Subject Headings (MeSH) terms and text words to maximize sensitivity. Search terms included, but were not limited to: “FBXO7” OR “F-box protein 7”; “PARK15”; “Parkinsonian-pyramidal syndrome” OR “pallido-pyramidal disease”. No language or geographic restrictions were applied during the initial search phase. Both single-case reports and larger patient cohorts (including family pedigrees and multicenter genetic screening studies) were collected for screening. To be eligible for final synthesis, studies had to meet the following predefined criteria: Inclusion Criteria—1, Confirmed molecular diagnosis with biallelic *FBXO7* variants (homozygous or compound heterozygous); 2, Availability of individual- or cohort-level clinical phenotype data; and 3, Pedigree architecture and/or segregation data consistent with autosomal recessive inheritance (though cases lacking complete segregation data were not strictly excluded if the variants were explicitly confirmed to be biallelic); Exclusion Criteria—1, Studies evaluating single heterozygous carriers without a second pathogenic variant on the opposite allele; 2, Purely in vitro or animal-model functional studies lacking human clinical data; and 3, Reviews, conference abstracts lacking detailed case data, and duplicate publications of the same patient cohorts.

Two independent reviewers screened titles and abstracts, followed by a full-text assessment of eligible articles. For each included case, a standardized data extraction template captured: age of onset, age at diagnosis, age at death (if any), geographical origin, consanguinity, Parkinsonian and pyramidal signs, and other clinical data (neuroimaging, other neurological signs, other systems involvement). Furthermore, genetic data were extracted for each case: genomic coordinates, nucleotide and amino acid changes, and variant types (e.g., missense, nonsense, splice-site, frameshift insertions/deletions). All reported genetic variants were re-annotated according to the NM_012179.4 (MANE) select transcript. Variant nomenclature was strictly standardized in accordance with the Human Genome Variation Society (HGVS) guidelines, ensuring an accurate and uniform comparison of the mutational spectrum across all historical cohorts.

Clinical features were collected, focusing attention on age at onset, the presence/absence of PPS signs and atypical manifestations, and the response to L-DOPA treatment. When available, additional data on geographic origin and neuroimaging findings (cranial MRI, brain iron accumulation) were also collected.

### 2.3. Data Integration and Analysis

Aggregated data from published cases and the newly identified family were integrated into a unified dataset. Variants were categorized by molecular consequence (missense, stop-gained, frameshift, and splicing), and further annotated for their functional impact (loss of function, impaired protein function, protein–protein interactions, and splicing defects). In addition, variants were mapped onto FBXO7 protein domains to explore their distribution across functionally relevant regions. Integrated data were then analyzed to investigate variant distribution, geographic patterns, and genotype–phenotype correlations.

## 3. Results

A total of 44 patients from 20 published studies were identified through the literature review, in addition to two affected individuals from a newly identified Italian family. Aggregated data were analyzed to characterize the mutational and clinical spectrum of *FBXO7*-related parkinsonism.

### 3.1. Clinical and Genetic Characterization of the Newly Identified Family

**Figure 1:**
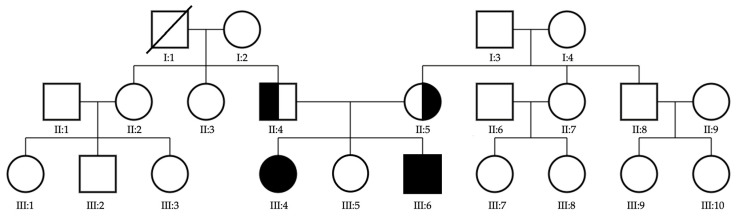
Pedigree of the family. Patients (III:4 and III:6) were born to non-consanguineous parents. No family members exhibited parkinsonism features or neurological disorders.

The family is of Italian origin, and the patients’ parents are not related. The evaluated family comprises two healthy parents, who gave birth to two affected children and one healthy child (Figure 1). The index cases were a female and a male, aged 26 and 19 years, respectively, at the first evaluation. The healthy daughter was evaluated at age 26.

Clinical findings of the two affected siblings are described below.

#### 3.1.1. Patient 1 (Female)

The patient was referred to our neurological center at age 26 for suspected iatrogenic Parkinsonism, which emerged following neuroleptic therapy for bipolar disorder/schizophrenia. Her treatment regimen included lithium, amitriptyline/perphenazine, and paliperidone. In the two years preceding hospital admission, she experienced behavioral disturbances with occasional aggressiveness, language impairment, and prominent visual and auditory hallucinations. Upon admission, the offending neuroleptic medications were systematically discontinued; however, her Parkinsonian features persisted, prompting further investigation. Her first comprehensive neurological clinical evaluation was performed at age 26. This initial baseline examination was unremarkably normal except for a conduction aphasia-type speech disorder, mild impairment of finger tapping (left > right), and subtle slowing of fine hand movements. Neuropsychological evaluation confirmed moderate-to-severe bradyphrenia and motor slowing, associated with language impairment, reduced efficiency of executive functions, fatuity, and anxiety. An EEG demonstrated frequent slow delta anomalies, occasionally triphasic, with diffuse expression and anterior predominance. A baseline cranial MRI was normal, as were abdominal ultrasonography, ECG, and standard laboratory analyses of the blood and urine.

While the initial differential diagnosis focused heavily on drug-induced parkinsonism, genetic testing ultimately revealed pathogenic *FBXO7* mutations. Following this diagnosis, the patient underwent a therapeutic trial of low-dose L-Dopa (100–200 mg/day). This yielded a significant initial improvement in her motor symptoms; however, no levodopa-induced dyskinesias were observed. Ultimately, levodopa had to be permanently discontinued due to a severe long-term worsening of her underlying behavioral disorders and emotional lability.

The patient was followed longitudinally at our center for a total duration of fourteen months. At her most recent clinical examination (age 27), she presented with mild bradykinesia, moderate bradyphrenia, ideomotor apraxia, and a non-fluent, effortful speech disorder characterized by anomias, phonemic paraphasias, a limited vocabulary, and short, incomplete sentences. Due to her psychiatric intolerance to dopaminergic medications, only neurorehabilitative therapy is currently ongoing. Repeat longitudinal cranial MRI or further neuroimaging evaluations were not performed during this timeframe. In terms of disease progression and current functional status, her motor decline has remained gradual; she remains fully ambulatory, exhibits no wheelchair dependence, and does not require assistance with her activities of daily living.

#### 3.1.2. Patient 2 (Male)

The younger brother of Patient 1 was referred to our neurological center for progressive psychomotor slowing and noticeable language disturbances. In the year prior to hospital admission, he experienced progressive speech impairment, motor slowing, severe anxiety, and marked social withdrawal. His initial neurological evaluation at age 19 recorded prominent hypomimia and bradykinesia. By age 20, a follow-up neurological evaluation revealed a more complex presentation: a conduction aphasia-type speech disorder, bradykinesia with axial rigidity, hypomimia, hyperreflexia in the lower limbs, and a mild bilateral impairment of finger tapping (left > right) and fine hand movements. Notably, his extrinsic ocular motility was characterized by fragmented and intrusive saccades. An EEG showed slow theta waves with a diffuse distribution, anterior predominance, and marked facilitation during drowsiness. Neuropsychological evaluation revealed a flat mood, situational anxiety, and mild-to-moderate motor slowing, though without apparent limitation in ideation. A baseline Cranial MRI was normal. An abdominal ultrasound revealed mild liver steatosis, which was subsequently confirmed by blood laboratory analyses; all other systemic laboratory lines were normal.

Mirroring his sister’s clinical management, this patient was placed on a therapeutic trial of levodopa. However, his clinical response to levodopa was poor, provided no meaningful motor benefit, was poorly tolerated, and did not induce dyskinesias. Consequently, the treatment was promptly discontinued.

Like his sibling, this patient was followed longitudinally for a targeted duration of fourteen months, and no repeat follow-up cranial MRIs were performed during this period. At his most recent examination (age 21), his clinical presentation consisted of mild bradykinesia, persistent axial rigidity, hypomimia, bradyphrenia, and expressive speech disorders. He is managed strictly with regular neurorehabilitative therapy. Regarding his current functional status and disease progression over this three-year window, the patient remains fully ambulatory, does not require a wheelchair, and requires only mild assistance with complex activities of daily living.

The two affected siblings and their family members were subjected to CES. The CES approach was preferred because the analysis was performed for clinical purposes. Over 95% of targeted regions were covered at ≥25×: the median target coverage was 39.5× (range 35–77×), and a mean of 97.4% of target bases were covered at ≥25× (details about target region coverage distribution are provided in Supplementary Figure S1). The output data of the CES of the family members met the requirements for the next step bioinformatic analysis. With the use of the Sophia DDM platform, compound heterozygous variants NM_012179.4:c.[222A>G] NP_036311.3:p.(Ile74Met) rs1011399377 and NM_012179.4:c.[698_703delins] NP_036311.3:p.(Val233GlufsTer8) in *FBXO7* were identified. The variants were inherited from the father and from the mother, respectively. Segregation analysis in the healthy daughter (II:2) revealed that neither of the two variants was inherited by her.

The NM_012179.4:c.[222A>G] variant was not reported in scientific literature or in variant databases (ClinVar, LOVD, OMIM, and HGMD). It was also extremely rare in gnomAD Exomes and Genomes (1 allele in 29116, with an allele frequency of 0.00003435), and no homozygous individuals were observed. The c.222A>G variant results in a missense substitution (p.Ile74Met), replacing isoleucine with methionine. Structural analysis of the wild-type FBXO7 protein using AlphaFold maps the p.Ile74Met substitution directly into the highly conserved Ubiquitin-Like (UBL) domain (Figure S2A). The wild-type Isoleucine at position 74 is a highly hydrophobic residue (Figure S2B). Thermodynamic stability calculations yielded a predicted free energy change (ΔΔG^Stability^) of −0.41 kcal/mol, indicating that the substitution significantly destabilizes the tertiary folding of the UBL domain. This structural disruption is predicted to impair the domain’s ability to mediate critical protein–protein interactions without completely destroying the overall protein scaffold. According to ACMG guidelines, this variant was classified as likely pathogenic based on moderate evidence (PM2, PM3, PP1, and PM1). The affected residue is evolutionarily conserved (PhyloP Primates score: 1.000; 241-way PhyloP: 2.811), and the substitution involves two hydrophobic, uncharged amino acids. Notably, the variant lies within the ubiquitin-like (UBL) domain, a functionally critical region involved in protein localization and enriched in pathogenic variants, supporting the upgrading of PM1 to strong evidence.

The NM_012179.4:c.[698_703delins] variant was not reported in scientific literature or in variant databases (ClinVar, LOVD, OMIM, and HGMD). It was also not reported in gnomAD Exomes and Genomes. The NM_012179.4:c.[698_703delinsAAGTTGAGCGGGGTGGAAGTTGAGCGGGGTGGAAGTTG] variant results in a complex indel causing a frameshift from codon 233, leading to a premature termination codon eight amino acids downstream (p.Val233GlufsTer8). This change is predicted to produce a truncated *FBXO7* protein lacking the entire C-terminal portion beyond residue 240. According to UniProt and InterPro annotations of FBXO7, this premature stop codon occurs within the annotated FP domain and is predicted to eliminate the downstream C-terminal regions, including the F-box domain (Figure S3). Therefore, this variant is predicted to cause loss of multiple conserved functional regions of FBXO7 and may result in a loss-of-function effect. According to ACMG guidelines, the variant was classified as likely pathogenic, supported by very strong evidence of pathogenicity (PVS1) and supporting evidence of pathogenicity (PM2). The very strong evidence for the PVS1 criterion was applied because it is a null variant (frameshift) that is expected to undergo nonsense-mediated decay or result in a severely truncated protein, consistent with loss-of-function as an established disease mechanism for *FBXO7*. Notably, the variant affects a region involved in protein dimerization and interaction with PSMF1, further supporting its functional relevance.

A limitation of the current study is the absence of wet-lab functional validation (such as protein stability or ubiquitination assays) in patient-derived cells, owing to sample availability constraints. To mitigate this and preliminarily evaluate the pathogenic mechanism of the novel variants, we performed in silico structural modeling and thermodynamic stability assessments. While these in silico data strongly support the likely pathogenic status of both variants, future in vitro functional assays in relevant cellular models remain necessary to definitively characterize their precise biochemical consequences.

### 3.2. Overview of Reported FBXO7 Cases

**Figure 2:**
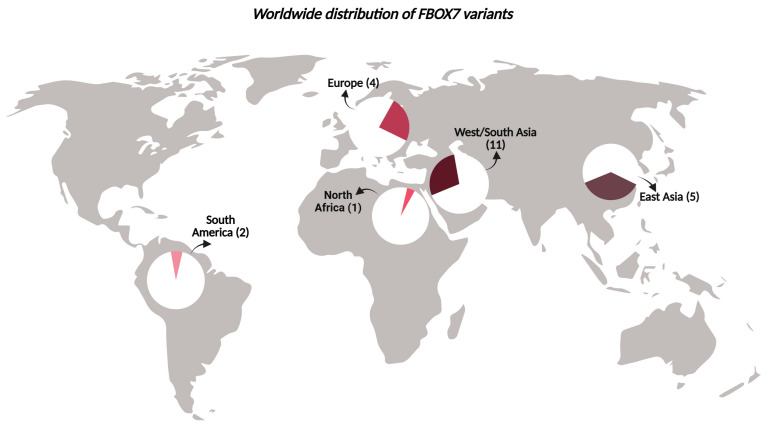
Worldwide distribution of *FBXO7* variants. Geographic distribution of *FBXO7* variants identified in previously reported cases and in the newly described family. Data are derived from the aggregated cohort summarized in Table 1. The world map illustrates the geographic distribution and relative proportions of *FBOX7* variants across different global regions. The numbers in parentheses indicate the total count of reported variants within each region. Colored pie sectors represent the specific regional proportion of *FBOX7* variants. White pie sectors represent the remaining proportion of *FBOX7* variants. This figure has been created with Biorender.

To provide a comprehensive and up-to-date overview of *FBXO7*-associated disease, all previously reported cases were systematically collected and integrated with the two additional patients (Table 1). Twenty-one articles were analyzed to extrapolate phenotype and genotype data. The aggregated cohort included patients from multiple geographic regions, although most reported cases originated from Europe, West Asia and East Asia (Figure 2).

In particular, four families are from Europe (1 from The Netherlands, 1 from Northeastern Europe, and 2 from Italy) [18,25], 10 from West Asia (1 from Iran, 7 from Turkey, 1 from Yemen, 1 from Israel) [7,11,12,13,15,16,19,20,22,24], 1 from South Asia (1 from Pakistan) [11], 5 from East Asia (3 from China, 2 from Korea) [6,8,9,10,21], 1 from Morocco [14], and 1 from Brazil [23]. However, this distribution likely reflects reporting and ascertainment biases rather than true differences in disease prevalence. The rarity of the condition, its autosomal recessive inheritance, and the higher likelihood of detection in consanguineous populations, together with differences in access to genetic testing and clinical expertise, may all contribute to the observed geographic pattern.

Clinical and instrumental findings were extracted from the literature and integrated with the detailed neurological evaluation of the newly described patients. Aggregated clinical features are summarized in Table 1 and further stratified according to variant type and zygosity (Table 2).

The most prevalent clinical features included Parkinsonian signs, particularly postural instability (87.5%), bradykinesia (83.3%), and rigidity (81.4%), as well as pyramidal signs (approximately 60% for all). Dysarthria is a clinical feature not frequently described, so the 76.5% frequency may be an overestimation of the real incidence.

Resting and action tremor, cognitive decline, and supranuclear gaze palsy are inconstant signs (with frequencies of 37.2%, 50%, 46.3%, and 32.1%, respectively). Similarly, cranial MRI abnormalities are variable and often become evident several years after the clinical onset of the disease.

All patients showed biallelic variants in the *FBXO7* gene. A total of 15 families (32 patients) were homozygous, and 10 families (14 patients) were compound heterozygous, although in some cases, segregation analysis was not available. The NM_012179.4:c.1492C>T variant (p.Arg498Ter) was the most frequently reported (in 13 patients), followed by the NM_012179.4:c.65C>T (p.Thr22Met). In the overall cohort of PARK15 patients, 12 families had affected individuals who were homozygous or compound heterozygous for a nonsense variant; 3 families had affected individuals who were homozygous for a missense variant; and 8 families had affected individuals who were compound heterozygous for a nonsense and a missense variant. Due to small sample sizes, no statistical comparison was performed; trends are hypothesis-generating only.

**Figure 3:**
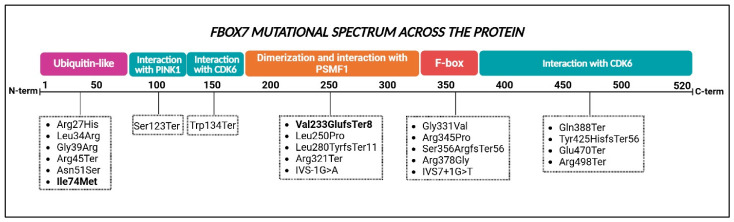
Distribution of *FBXO7* variants across protein domains. Schematic representation of the FBXO7 protein with functional domains and regions of interest annotated according to UniProt (Q9Y3I1). Reported variants are mapped based on their position and include those identified in published cases and in the newly described family (in bold). This figure has been created with Biorender.

**Figure 4:**
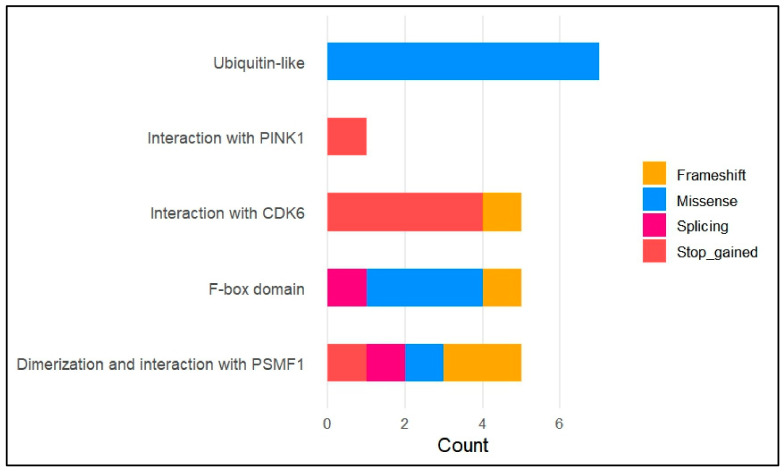
Distribution of *FBXO7* variants by molecular consequence across protein domains. Bar plot showing the number of *FBXO7* variants across functional domains, stratified by molecular consequence. The distribution highlights the presence of both missense and truncating variants across different regions of the protein.

The spectrum of *FBXO7* variants identified in the aggregated cohort is summarized in Table 3. Variants include missense, truncating (stop-gained and frameshift), and splicing alterations. Mapping of variants across the FBXO7 protein (Figure 3) revealed a clustering of missense variants within the N-terminal UBL domain and to a lesser extent in the F-box domain (Figure 4), both critical for protein function and protein–protein interactions. Truncating variants were more frequently observed in the central and C-terminal regions of the protein, affecting regions implicated in dimerization and interaction with PINK1, PSMF1 or CDK6 (Figure 4). This pattern supports the presence of distinct molecular mechanisms underlying *FBXO7*-related parkinsonism, including loss-of-function and altered protein function affecting protein–protein interactions, depending on variant localization.

## 4. Discussion

By integrating two newly identified cases with all previously reported variants, we delineate the mutational and clinical landscape of *FBXO7*-related parkinsonism. A non-random spatial distribution of variants emerges across FBXO7 functional regions: missense changes cluster preferentially within the N-terminal UBL and F-box domains, while truncating and splicing variants predominate in the central and C-terminal portions of the protein (Figure 4).

The *FBXO7* gene encodes a multifunctional protein acting as a subunit of the SCF (Skp1-Cullin1-F-box) E3 ubiquitin ligase complex. This complex tags specific substrates for proteasomal degradation and mitochondrial quality control. The FBXO7 protein contains several critical domains and regions of interest for its functional activity. Through its UBL domain located at the N-terminus, FBXO7 physically interacts with both PINK1 and Parkin to regulate proteasome binding. This interaction promotes Parkin’s translocation from the cytosol to damaged mitochondria. The F-box domain links the protein to the SCF complex. Furthermore, FBXO7 helps stabilize PINK1 and may assist the SCF complex in ubiquitinating mitochondrial substrates such as Mitofusin 1 (Mfn1). In addition, specific regions of the protein mediate dimerization and interaction with PSMF1/PI31 [7,26], a key regulator of proteasome activity, as well as binding to CDK6, which is implicated in a diverse set of cellular processes and neuronal death processes [27]. Disruption of these functions may lead to impaired mitophagy, defective proteasomal activity, and accumulation of damaged mitochondria and misfolded proteins, ultimately contributing to neurodegeneration. The distribution of variants observed in this study is consistent with these functional roles: missense variants may preferentially impair protein interactions and subcellular localization, whereas truncating variants are more likely to result in complete loss of function, affecting both proteasomal and mitochondrial pathways.

From a genetic perspective, *FBXO7*-related parkinsonism is characterized by a wide mutational spectrum (Figure 3), with most variants being private or family-specific and only a few recurrent changes. To date, 23 causative *FBXO7* mutations (8 missense, 13 nonsense, and 2 splicing) have been reported in the literature, with only one recurrent variant (p.Arg498Ter) identified in multiple families (Table 3). The geographic distribution (Figure 2) of reported cases is heterogeneous and likely reflects ascertainment biases and differences in access to genetic testing rather than true population-specific prevalence. The autosomal recessive inheritance pattern further contributes to the increased frequency of cases in populations with higher rates of consanguinity. The global distribution (Figure 2) is shaped by a combination of both artifactual and underlying biological factors. On one hand, reporting and ascertainment biases strongly skew the mapped distribution due to uneven international access to clinical exome/genome sequencing, localized research expertise, and a distinct publication bias toward novel variants in high-income centers. On the other hand, true biological and population-genetic mechanisms drive these geographic patterns. The marked concentration of cases within specific West Asian regions reflects a high coefficient of inbreeding; because PARK15 is an autosomal recessive condition, a high frequency of parental consanguinity naturally maximizes the clinical occurrence of rare recessive disorders.

Furthermore, regional clusters are heavily influenced by founder effects and population-restricted variants rather than uniform global prevalence. For example, the nonsense variant p.Arg498Ter serves as a recurring molecular signature across multiple unrelated families of distinct demographic origins (Italy, Pakistan, and Turkey), leading to a highly localized presentation of the disease phenotype. Otherwise, the p.Thr22Met variant has been documented on different continents (Europe and South America).

Ultimately, these geographical patterns underscore the need for expanded global sequencing efforts and comprehensive haplotype analyses. Collaborative international genomic registries that track diverse cohorts are essential to definitively characterize these ancestral lineages, identify novel localized founder effects, and map the true global epidemiology of *FBXO7*-related parkinsonism.

Moreover, 493 *FBXO7* variants have been listed in public databases such as ClinVar, of which 49 have been classified as Pathogenic/Likely pathogenic and 10 have conflicting pathogenicity classifications, highlighting the challenges in variant interpretation.

Clinically, *FBXO7*-related disease is typically characterized by a PPS, although significant phenotypic variability exists across families (Table 1). This clinical heterogeneity may tentatively reflect the underlying molecular diversity, with different classes of variants potentially affecting distinct functional domains of the protein (Figure 3). Patients with biallelic nonsense variants exhibited the most complete phenotype, with Parkinsonian signs being almost invariably present and pyramidal signs occurring in approximately 50% of cases. Conversely, subjects with biallelic missense variants showed a higher incidence of pyramidal signs (>80%), but a lower frequency of Parkinsonian features (<50%). Because of the very small sample size, formal statistical comparisons were not performed, and these descriptive observations cannot achieve statistical significance. Patients carrying compound heterozygous missense/nonsense variants appeared to display an intermediate pattern, with frequencies of both Parkinsonian and pyramidal signs falling between those observed in the two homozygous groups, although the limited number of cases remains a major constraint. Consequently, these trends must be interpreted with caution as strictly preliminary and hypothesis-generating. They are intended to serve as a baseline for future validation through formal genotype–phenotype correlations in larger cohorts as they become available. A descriptive summary of all reported PARK15 patients is provided in Table 1.

Although long-standing documentation of clinical phenotypes in PARK15 patients remains scarce in the scientific literature, we summarized the available presentation patterns to explore whether these cases might fit into established Parkinson’s disease subtypes [28]. Out of the 46 patients, only 10 exhibited descriptive characteristics suggesting alignment with one of the four clinical subtypes (benign tremulous parkinsonism, PD-dementia, PD-axial dystonia, and PD non-motor or paucimotor subtypes). Specifically, three cases could tentatively be categorized within the Parkinson’s dementia group [7,8,11]; notably, these cases lacked molecular similarities to support a common underlying mechanism. Furthermore, intrafamilial variability was highlighted by one case presenting with a phenotype distinct from family members who could not be classified into any subtype [11]. At a descriptive level, two cases aligned with benign tremulous parkinsonism [19,24]. Three sporadic cases fell into the non-motor/pauci-motor parkinsonism subtype [10,14,16], precluding an assessment of intrafamilial variability. Finally, in our family, Patient 1 and Patient 2 showed preliminary features suggestive of a pauci-motor PD subtype. Given the limited data, longer clinical follow-up is essential to validate or refute this preliminary categorization. A less frequent and highly variable clinical feature was the presence of ophthalmological signs [7,11,14]. Due to their inconsistency, these signs may be related to other genetic factors, particularly as they became apparent only in patients with a history of consanguinity.

Comparison with other autosomal recessive forms of parkinsonism highlights both shared and distinct features. The two most common forms of autosomal recessive Parkinson’s disease, PARK2 (Parkin) and PARK6 (PINK1), share a clinical presentation and slow progression but are generally distinguished from PARK15 by the high frequency of limb dystonia and psychiatric signs. Similarly, PARK7 (DJ1) presents with early onset and psychiatric or behavioral symptoms. Conversely, PARK9 (ATP13A2), PARK14 (PLA2G6), and PARK23 (VPS13C) are typically complicated by cognitive impairment, sometimes with neuroradiological signs (such as NBIA and cerebellar atrophy in PARK14). While all conditions manifest with extrapyramidal signs, *FBXO7*-related disease is uniquely characterized by its “atypical” clinical features. In fact, pyramidal signs are typical in PARK15, rare in PARK23, PARK9, and PARK14, and absent in PARK2 and PARK6 cases.

At the molecular level, *FBXO7* appears to act as a modulator of the PINK1/Parkin pathway while also playing an independent role in proteasomal regulation, suggesting a broader functional impact compared to PRKN and PINK1. These proteins can be viewed as part of a shared pathway involved in mitochondrial quality control, although their specific roles differ. PINK1 and Parkin represent the core machinery driving mitophagy, whereas FBXO7 is thought to facilitate this process by promoting Parkin recruitment to damaged mitochondria through its UBL domain. In contrast to PINK1 and Parkin, FBXO7 is also a component of the SCF E3 ubiquitin ligase complex and plays a role in proteasomal assembly and activity. This dual function suggests that *FBXO7*-related disease may involve a broader disruption of cellular homeostasis, extending beyond mitophagy to include proteasomal dysfunction and altered protein turnover.

While some studies challenge an essential role for FBXO7 in PINK1/Parkin-mediated mitophagy, converging evidence from patient-derived models and in vivo studies supports a modulatory function, particularly in setting ubiquitination thresholds and stabilizing PINK1 [7,29,30,31]. The phenotypic overlap with PARK2/PARK6 may therefore reflect convergent pathway disruption rather than identical molecular mechanisms [32]. These findings highlight the need to consider additional pathogenic mechanisms beyond mitophagy, including proteasomal dysfunction and dysregulation of cell cycle-related pathways, which may contribute to disease pathogenesis and distinguish PARK15 from other forms of autosomal recessive parkinsonism. Taken together, our data support a model in which both complete loss of function and domain-specific disruptions converge to produce the PARK15 phenotype distinctions with tangible implications for variant counseling and, ultimately, for stratifying patients who might benefit from targeted therapeutic strategies.

## 5. Conclusions

Collectively, this descriptive synthesis of PARK15 cases offers preliminary insights that may help shape future mechanistic hypotheses linking variant class and domain localization to clinical trends. While core motor features—such as postural instability (87.5%), bradykinesia (83.3%), and rigidity (81.4%)—appear nearly universal in this cohort, the frequent descriptive co-occurrence of pyramidal signs (~60%), early cognitive decline (46.3%), and psychiatric manifestations highlights features that may characterize the PARK15 phenotype. Based on these observations, we hypothesize that truncating variants, which are predicted to abolish FBXO7 function entirely, might be associated with earlier disease onset and a broader phenotypic burden. Conversely, domain-specific missense substitutions-particularly within the UBL domain-might selectively disrupt specific protein–protein interactions rather than eliminating the entire functional scaffold. This tentative distinction represents a hypothesis-generating framework; future studies will need to explore whether patients with potential residual FBXO7 activity display distinct responses to interventions aimed at modulating mitophagy or proteasomal flux.

Several open questions emerge from this work and warrant dedicated follow-up. The cellular consequences of UBL-domain missense substitutions—such as the p.Ile74Met variant described here—remain to be established in physiologically relevant neuronal models. Patient-derived iPSC dopaminergic neurons offer a valuable potential platform to investigate whether the primary driver of neurodegeneration involves impaired mitophagy, defective proteasomal assembly, or dysregulated CDK6-mediated cell cycle re-entry, as these questions cannot be resolved from clinical data alone. Concurrently, the rarity of PARK15 worldwide—compounded by potential ascertainment bias and uneven access to genomic diagnostics—highlights the need for coordinated international registries linking molecular data to longitudinal clinical trajectories. Such collaborative efforts would provide the statistical power necessary to formally test the genotype–phenotype trends outlined here and to identify potential early biomarkers of disease progression. From a translational standpoint, the role of FBXO7 at the intersection of mitochondrial quality control and proteasomal regulation makes it an interesting candidate node for future pharmacological exploration. Speculatively, small molecules capable of stabilizing PINK1 or enhancing proteasomal activity without globally disrupting the ubiquitin-proteasome system could be investigated to determine if they can partially compensate for FBXO7 loss of function in models retaining residual protein activity. Furthermore, the corticospinal tract pathology underlying the pyramidal component of PARK15—a feature generally absent from PARK2 and PARK6—warrants dedicated neuropathological investigation to explore a potential vulnerability of upper motor neurons to disrupted protein homeostasis. In clinical practice, the presence of pyramidal signs and early cognitive decline may serve as preliminary diagnostic red flags warranting the consideration of prompt molecular testing in a young patient whose parkinsonism deviates from the typical idiopathic Parkinson’s disease profile. Ultimately, a broader implementation of clinical exome or genome sequencing, combined with systematic re-annotation of *FBXO7* variants against the MANE reference transcript, remains essential to help shorten the diagnostic odyssey that currently affects most PARK15 families.

## Figures and Tables

**Table 1:** Clinical and imaging features of PARK15 patients. F: female; M: male; n.a.: not available.

		Infancy (0–1 Year)	Childhood (1–11 Years)	Adolescence (12–17 Years)	Young Adulthood (18–40 Years)	Middle Age (41–60 Years)	Total
No. of patients (gender percentage)		3 (33% F, 67% M)	2 (100% F)	12 (42% F, 58% M)	24 (42% F, 58% M)	5 (20% F, 80% M)	46 (41% F, 59% M)
age at onset (min–max)		5.6 mo (5 mo–6 mo)	10	14.8 (12–17)	24.2 (18–31)	45 (41–52)	23 (5 mo–52)
age at last examination		5.5 (5–6)	14	24 (14–39)	32.7 (21–45)	51.4 (43–62)	30 (5–62)
age at death		2 * (1 case)	n.a.	26 (24–28) * (2 cases)	n.a.	n.a.	(2–28)
parkinsonism	bradykinesia	n.a.	2/2	12/12	16/23	5/5	35/42 (83.3%)
	rigidity	3/3	2/2	12/12	14/21	4/5	35/43 (81.4%)
	postural instability	n.a.	2/2	8/9	8/10	3/3	21/24 (87.5%)
	(resting) tremor	1/1	1/2	7/12	4/23	3/5	16/43 (37.2%)
	action tremor	n.a.	1/1	5/6	6/17	n.a.	12/24 (50%)
pyramidal signs	Spastic paraplegia	n.a.	1/2	4/8	14/17	0/3	19/30 (63.3%)
	hyperreflexia	2/2	1/1	7/11	14/21	0/5	24/40 (60%)
	Babinski sign	1/1	1/1	5/11	14/21	1/4	22/38 (57.9%)
others	cognitive decline/dementia	3/3	1/2	10/12	4/19	1/5	19/41 (46.3%)
	supranuclear gaze palsy	n.a.	0/1	5/8	4/18	0/1	9/28 (32.1%)
	dysarthria	n.a.	2/2	7/7	2/6	2/2	13/17 (76.5%)
	brain iron accumulation	0/1	0/2	2/12	1/10	0/2	3/27 (11.1%)
	Cranial MRI abnormal	1/3	0/2	6/12	4/13	2/5	13/35 (37.1%)
References		[7,21]	[12,18]	[8,11,12,13,14,15,16,18,22]	[6,9,11,17,18,19,23], our case	[10,24,25]	

* The age at death is calculated only for the cases reported as deaths.

**Table 2:** Clinical and imaging features of PARK15 patients aggregated for genotype. F: female; M: male; n.a.: not available.

		Biallelic for a Missense Variant	Compound Heterozygous Missense/Nonsense Variants	Biallelic for a Nonsense Variant
No. of patients (gender percentage)		15 (33% F, 67% M)	14 (36% F, 64% M)	17 (53% F, 47% M)
age at onset (min–max)		38 (5 mo–52)	27 (16–45)	15 (6 mo–24)
age at last examination		40 (5–62)	34 (21–51)	35 (6–40)
age at death		26 *	2 *	n.a.
parkinsonism	bradykinesia	7/14	13/13	15/15
	rigidity	8/15	10/11	17/17
	postural instability	3/3	8/11	10/10
	(resting) tremor	3/15	6/13	7/15
	action tremor	1/12	6/6	5/6
pyramidal signs	Spastic paraplegia	10/12	2/6	7/12
	hyperreflexia	12/15	4/13	8/12
	Babinski sign	14/14	3/13	5/11
others	cognitive decline/dementia	2/14	3/10	14/17
	supranuclear gaze palsy	0/11	2/6	7/11
	Dysarthria ^(1)^	2/2	1/5	10/10
	brain iron accumulation	0/6	1/6	2/15
	Cranial MRI abnormal ^(2)^	3/7	3/11	7/17
References		[6,7,20,24]	[8,9,10,17,18,19,23,25], our case	[11,12,13,14,15,16,18,21,22]

* The age at death is calculated only for the cases reported as deaths. ^(1)^ Sign reported in only a subset of studies; likely inflated by ascertainment bias. ^(2)^ Neuroradiological abnormalities reported are: General atrophy [8,11], cerebral atrophy [13], cortical atrophy and periventricular white matter lesions [24], cerebral and brainstem atrophy [16], vermian atrophy progressing to cerebral and cerebellar atrophy with brain iron deposition in the pallidum and substantia nigra [14], hippocampal and cortical atrophy with ischemic lesions, cerebral atrophy and reduced glucose metabolism in temporal and parietal lobes [6], severe global cortical atrophy, mesencephalic atrophy, tetraventricular hydrocephalus and iron accumulation [15], frontotemporal lobe atrophy with iron deposition in the globus pallidum and substantia nigra, and hypometabolism in frontal and temporoparietal regions [17], bilateral hypointensities in pallidum in the SWI sequence [10], and thin corpus callosum [7].

**Table 3:** *FBXO7* variants associated with PARK15. Summary of *FBXO7* variants currently known to be associated with the disease. The novel variants reported in this study have been highlighted in bold. Nucleotide and protein coding of variants refers to NM_012179.4 and NP_036311.3. Protein localization was defined based on functional domains and regions of interest annotated in UniProt (Q9Y3I1).

Nucleotide Coding	Protein Coding	N° Times Identified	Variant Type	Exon/Intron	Protein Localization	Functional Impact
c.65C>T	p.(Thr22Met)	2	missense	1	Ubiquitin-like	Impaired protein function
c.80G>A	p.(Arg27His)	2	missense	1	Ubiquitin-like	Impaired protein function
c.101T>G	p.(Leu34Arg)	1	missense	1	Ubiquitin-like	Impaired protein function
c.115G>A	p.(Gly39Arg)	1	missense	1	Ubiquitin-like	Impaired protein function
c.133C>T	p.(Arg45Ter)	1	stop-gained	2	Ubiquitin-like	Impaired protein function
c.152A>G	p.(Asn51Ser)	1	missense	2	Ubiquitin-like	Impaired protein function
**c.222A>G**	p.(Ile74Met)	1	missense	2	Ubiquitin-like	Impaired protein function
c.368C>G	p.(Ser123Ter)	1	stop-gained	2	Important for interaction with PINK1	Loss of function
c.402G>A	p.(Trp134Ter)	1	stop-gained	2	Important for interaction with CDK6	Loss of function
**c.698_703delins**	p.(Val233GlufsTer8)	1	frameshift	4	Important for dimerization and interaction with PSMF1	Loss of function
c.749T>C	p.(Leu250Pro)	1	missense	4	Important for dimerization and interaction with PSMF1	Impaired protein function
c.838del	p.(Leu280TyrfsTer11)	1	frameshift	5	Important for dimerization and interaction with PSMF1	Loss of function
c.872-1G>A	-	1	splicing	IV5	Important for dimerization and interaction with PSMF2	Loss of function (splicing defect)
c.961C>T	p.(Arg321Ter)	1	stop-gained	6	Important for dimerization and interaction with PSMF1	Loss of function
c.992G>T	p.(Gly331Val)	1	missense	7	F-box domain	Impaired protein function
c.1034G>C	p.(Arg345Pro)	1	missense	7	F-box domain	Impaired protein function
c.1066_1069del	p.(Ser356ArgfsTer56)	1	frameshift	7	F-box domain	Loss of function
c.1132C>G	p.(Arg378Gly)	1	missense	7	F-box domain	Impaired protein function
c.1144+1G>T	-	1	splicing	IV7	F-box domain	Loss of function (splicing defect)
c.1162C>T	p.(Gln388Ter)	1	stop-gained	8	Important for interaction with CDK6	Loss of function
c.1268_1272dup	p.(Tyr425HisfsTer56)	1	frameshift	9	Important for interaction with CDK6	Loss of function
c.1408G>T	p.(Glu470Ter)	1	stop-gained	9	Important for interaction with CDK6	Loss of function
c.1492C>T	p.(Arg498Ter)	13	stop-gained	9	Important for interaction with CDK6	Loss of function

## Data Availability

The original contributions presented in this study are included in the article/Supplementary Materials. Further inquiries can be directed to the corresponding author.

## Supplementary Materials

The following supporting information can be downloaded at: https://www.mdpi.com/article/10.3390/genes17070764/s1, Table S1: primers used for sequencing. Table S2: Levodopa response in PARK15 patients. Figure S1: Target region coverage distribution for samples II:4, II:5, III:4, and III:6. Figure S2. A: Structural analysis of the wild-type FBXO7 protein (AlphaFold AF-Q9Y3I1-F1). B: Interactions of the I74 residue within the FBXO7 protein (DynaMut2). Figure S3. Predicted effect of NM_012179.4:c.[698_703delins] variant on NP_036311.3. A: domain architecture of wild-type and truncated protein. B: Structural models of wild-type and truncated protein. References [6,7,8,9,10,11,12,13,14,15,16,17,18,19,20,21,22,23,24,25] are cited in Supplementary Materials.

## Abbreviations

The following abbreviations are used in this manuscript:

PPS	Parkinsonian-Pyramidal Syndrome

## References

[1] Davison C. (1954). Pallido-pyramidal disease. J. Neuropathol. Exp. Neurol..

[2] Moller J.C., Oertel W.H., Koller W.C., Melamed E. (2007). Other degenerative processes. Parkinson’s Disease and Related Disorders, Part II: Handbook of Clinical Neurology.

[3] Sutton J.P., Pulst S.-M. (2003). Other adult-onset movement disorders with a genetic basis. Genetics of Movement Disorders.

[4] Nisipeanu P., Kuritzky A., Korczyn A.D. (1994). Familial levodopa-responsive parkinsonian-pyramidal syndrome. Mov. Disord..

[5] Lunati A., Lesage S., Brice A. (2018). The genetic landscape of Parkinson’s disease. Rev. Neurol..

[6] Wang Z., Song Y., Zhu W., Wang X., Li X., Xu F., Si L., Yao T., Zhu J., Lai H. (2021). A novel FBXO7-R345P mutation in a Chinese family with autosomal recessive parkinsonian-pyramidal syndrome. Park. Relat. Disord..

[7] Al Rawi S., Simpson L., Agnarsdóttir G., McDonald N.Q., Chernuha V., Elpeleg O., Zeviani M., Barker R.A., Spiegel R., Laman H. (2024). Study of an FBXO7 patient mutation reveals Fbxo7 and PI31 co-regulate proteasomes and mitochondria. FEBS J..

[8] Wei L., Ding L., Li H., Lin Y., Dai Y., Xu X., Dong Q., Lin Y., Long L. (2018). Juvenile-onset parkinsonism with pyramidal signs due to compound heterozygous mutations in the F-Box only protein 7 gene. Park. Relat. Disord..

[9] Yoo D., Choi J.H., Im J.H., Kim M.J., Kim H.J., Park S.S., Jeon B. (2020). Young-Onset Parkinson’s Disease with Impulse Control Disorder Due to Novel Variants of F-Box Only Protein 7. J. Mov. Disord..

[10] Kim E.Y., Kim S.Y., Seo Y., Shin C. (2022). Nearly Abolished Dopamine Transporter Uptake in a Patient With a Novel FBXO7 Mutation. J. Mov. Disord..

[11] Paisán-Ruiz C., Guevara R., Federoff M., Hanagasi H., Sina F., Elahi E., Schneider S.A., Schwingenschuh P., Bajaj N., Emre M. (2010). Early-onset L-dopa-responsive parkinsonism with pyramidal signs due to ATP13A2, PLA2G6, FBXO7 and spatacsin mutations. Mov. Disord..

[12] Yalcin-Cakmakli G., Olgiati S., Quadri M., Breedveld G.J., Cortelli P., Bonifati V., Elibol B. (2014). A new Turkish family with homozygous FBXO7 truncating mutation and juvenile atypical parkinsonism. Park. Relat. Disord..

[13] Gündüz A., Eken A.G., Bilgiç B., Hanagasi H.A., Bilgüvar K., Günel M., Başak A.N., Ertan S. (2014). FBXO7-R498X mutation: Phenotypic variability from chorea to early onset parkinsonism within a family. Park. Relat. Disord..

[14] Correa-Vela M., Lupo V., Montpeyó M., Sancho P., Marcé-Grau A., Hernández-Vara J., Darling A., Jenkins A., Fernández-Rodríguez S., Tello C. (2020). Impaired proteasome activity and neurodegeneration with brain iron accumulation in FBXO7 defect. Ann. Clin. Transl. Neurol..

[15] Şahin E., Samanci B., Çakmaklı G.Y., Lohmann E., Güven G., Gökalp E.E., Gündüz A., Başak A.N., Ertan S., Elibol B. (2025). FBXO7 Pathogenic Variants in Early-Onset Parkinsonism: Insights from a Neuroimaging Perspective and Review of the Literature. Mov. Disord. Clin. Pract..

[16] Conedera S., Apaydin H., Li Y., Yoshino H., Ikeda A., Matsushima T., Funayama M., Nishioka K., Hattori N. (2016). FBXO7 mutations in Parkinson’s disease and multiple system atrophy. Neurobiol. Aging.

[17] Mahajan S., Mehta S., Singh J., Mehta S., Lal V. (2024). Parkinsonism with prominent neuropsychiatric symptoms without pyramidal involvement in a patient with FBXO7 variants. Neurol. Sci..

[18] Di Fonzo A., Dekker M., Montagna P., Baruzzi A., Yonova E.H., Guedes L.C., Szczerbinska A., Zhao T., Dubbel-Hulsman L.O., Wouters C.H. (2009). FBXO7 mutations cause autosomal recessive, early-onset parkinsonian-pyramidal syndrome. Neurology.

[19] Lorenzo-Betancor O., Lin Y.-H., Samii A., Jayadev S., Kim H.M., Longfellow K., Distad B.J., Yearout D., Mata I.F., Zabetian C.P. (2020). Novel compound heterozygous FBXO7 mutations in a family with early onset Parkinson’s disease. Park. Relat. Disord..

[20] Shojaee S., Sina F., Banihosseini S.S., Kazemi M.H., Kalhor R., Shahidi G.-A., Fakhrai-Rad H., Ronaghi M., Elahi E. (2008). Genome-wide linkage analysis of a Parkinsonian-pyramidal syndrome pedigree by 500 K SNP arrays. Am. J. Hum. Genet..

[21] Jin X., An L., Hao S., Liu Q., Zhang Q., Wang X., Feng X., Zhang C., Cao X., Yan Y. (2020). Compound heterozygous variants of the FBXO7 gene resulting in infantile-onset Parkinsonian-pyramidal syndrome in siblings of a Chinese family. J. Clin. Lab. Anal..

[22] Hanagasi H.A., Lees A., Johnson J.O., Singleton A., Emre M. (2007). Smoking-responsive juvenile-onset Parkinsonism. Mov. Disord..

[23] Lorenzo-Betancor O., Mehta S., Ramchandra J., Mumuney S., Schumacher-Schuh A.F., Cornejo-Olivas M., Sarapura-Castro E.H., Torres L., Inca-Martinez M.A., Mazzetti P. (2024). Parkinson’s Disease Gene Screening in Familial Cases from Central and South America. Mov. Disord..

[24] Lohmann E., Coquel A., Honoré A., Gurvit H., Hanagasi H., Emre M., Leutenegger A.L., Drouet V., Sahbatou M., Guven G. (2015). A new F-box protein 7 gene mutation causing typical Parkinson’s disease. Mov. Disord..

[25] Sarmiento I.J.K., Afshari M., Kinsley L., Silani V., Akhtar R.S., Simuni T., Lubbe S.J., Krainc D., Mencacci N.E. (2022). Novel bi-allelic FBXO7 variants in a family with early-onset typical Parkinson’s disease. Park. Relat. Disord..

[26] Magrinelli F., Tesson C., Angelova P.R., Rodriguez J.A., Scardamaglia A., O’Callaghan B., Lowe S.A., Salazar-Villacorta A., Chung B.H., Jaconelli M. (2025). Variants in the proteasome regulator PSMF1 cause a phenotypic spectrum from early-onset Parkinson’s disease to perinatal lethality and disrupt mitochondrial function. medRxiv.

[27] Deng H., Liang H., Jankovic J. (2013). F-box only protein 7 gene in parkinsonian-pyramidal disease. JAMA Neurol..

[28] Filidei M., Marsili L., Colosimo C. (2025). Do Parkinson’s Disease clinical subtypes really exist?. Neurol. Neurochir. Pol..

[29] Huang T., Fang L., He R., Weng H., Chen X., Ye Q., Qu D. (2020). Fbxo7 and Pink1 play a reciprocal role in regulating their protein levels. Aging.

[30] Liu Y., Lear T.B., Verma M., Wang K.Z., Otero P.A., McKelvey A.C., Dunn S.R., Steer E., Bateman N.W., Wu C. (2020). Chemical inhibition of FBXO7 reduces inflammation and confers neuroprotection by stabilizing the mitochondrial kinase PINK1. JCI Insight.

[31] Sanchez-Martinez A., Martinez A., Whitworth A.J. (2023). FBXO7/ntc and USP30 antagonistically set the ubiquitination threshold for basal mitophagy and provide a target for Pink1 phosphorylation in vivo. PLoS Biol..

[32] Kraus F., Goodall E.A., Smith I.R., Jiang Y., Paoli J.C., Adolf F., Zhang J., Paulo J.A., Schulman B.A., Harper J.W. (2023). PARK15/FBXO7 is dispensable for PINK1/Parkin mitophagy in iNeurons and HeLa cell systems. EMBO Rep..

