# Supplementary material for: Expanding the Clinical and Mutational Spectrum of FBXO7-Related Parkinsonism: A Novel Italian Family and Comprehensive Literature Review
Source: Genes (Basel). 2026 Jun 30;17(7):764. doi: 10.3390/genes17070764 (PMC13408918; doi:10.3390/genes17070764)

Table S1: Primers used for sequencing.

	Sequence (5'->3')
**Forward primer – exon 2**	GCTTCAGATGTGCTATTGAAGGT
**Reverse primer – exon 2**	TCCTGACTGGAACAGAATTCAACT
**Product length – exon 2**	448
**Tm – exon 2**	59
**Forward primer – exon 4**	AGAGGACTGTGTGGAGTGATTA
**Reverse primer – exon 4**	GCCAATCACTGAAGTGGCTATAAA
**Product length – exon 4**	343
**Tm – exon 4**	58-59

Table S2: Levodopa response in PARK15 patients. F: female; M: male; n.a.: not available. References cited are the same as in the main manuscript.

Gender	Age at onset	Age at last examination	Age at death	Levodopa response	Reference
M	third decade	n.a.	n.a.	good	[20]
F	10	n.a.	n.a.	good with dyskinesia and psychiatric features	[18]
M	13	n.a.	n.a.	good with dyskinesia and psychiatric features	[18]
F	18	n.a.	n.a.	good with dyskinesia and psychiatric features	[18]
M	19	n.a.	n.a.	good with dyskinesia and psychiatric features	[18]
F	17	22	n.a.	good with psychiatric features	[11]
F	24	40	n.a.	good	[11]
F	22	n.a.	n.a.	n.a.	[11]
M	17	26	28	good with psychiatric features	[11,22]
M	14	21	n.a.	poor with dyskinesia	[12]
F	10	14	n.a.	poor with psychiatric	[12]
M	13	14	n.a.	good with psychiatric features	[13]
F	17	17	24	good	[13]
F	52	62	n.a.	good	[24]
M	41	52	n.a.	good	[24]
F	13	39	n.a.	good with dyskinesia and psychiatric features	[16]
M	16	32	n.a.	good with psychiatric features	[8]
M	6 months	6	n.a.	n.a.	[21]
M	6 months	n.a.	2	poor	[21]
M	28	31	n.a.	good with psychiatric features	[9]
F	21	30	n.a.	good with psychiatric features	[19]
F	27	33	n.a.	good with dyskinesia	[19]
M	30	33	n.a.	n.a.	[19]
F	12	21	n.a.	poor	[14]
M	30	37	n.a.	poor	[6]
F	31	45	n.a.	good	[6]

M	42	43	n.a.	poor with psychiatric features	[10]
M	45	49	n.a.	good	[25]
M	45	51	n.a.	good	[25]
F	29	n.a.	n.a.	n.a.	[23]
M	16	21	n.a.	good with psychiatric features	[15]
M	15	21	n.a.	poor	[15]
F	15	30	n.a.	poor	[15]
M	18	30	n.a.	good with psychiatric features	[17]
F	5 months	5	n.a.	good	[7]
M	18	21	n.a.	good with psychiatric features	present case
F	24	27	n.a.	good with psychiatric features	present case

Figure S1. Target region coverage distribution for samples II:4, II:5, III:4, and III:6.

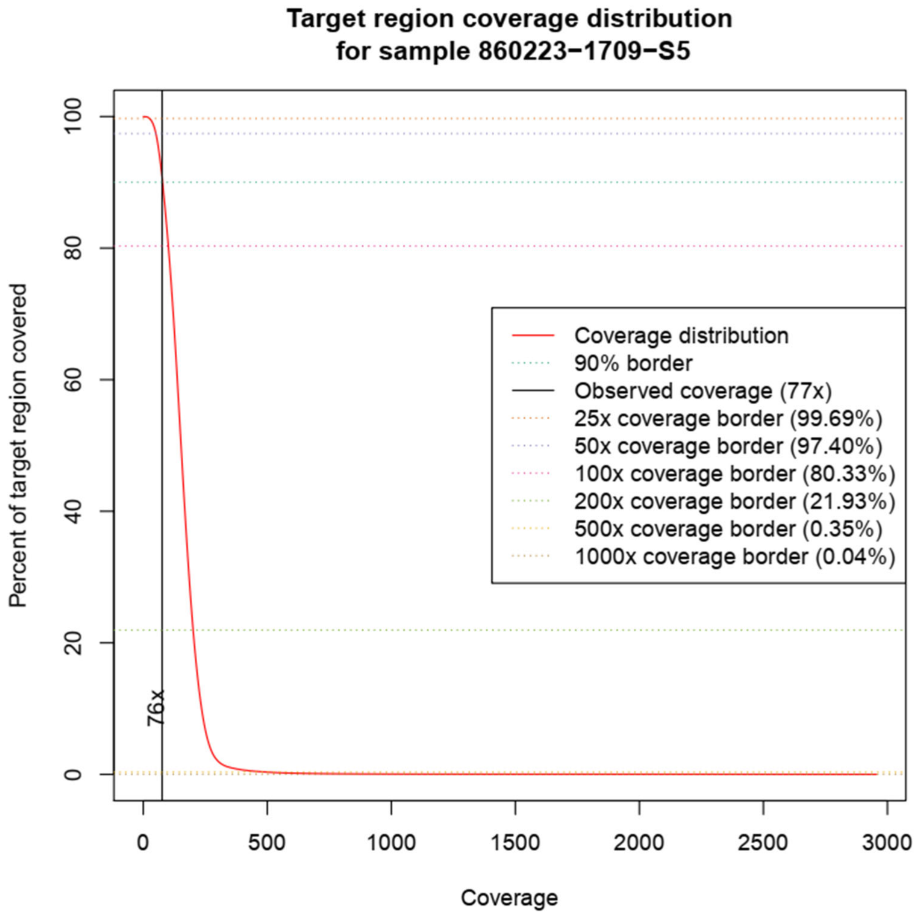

II:4

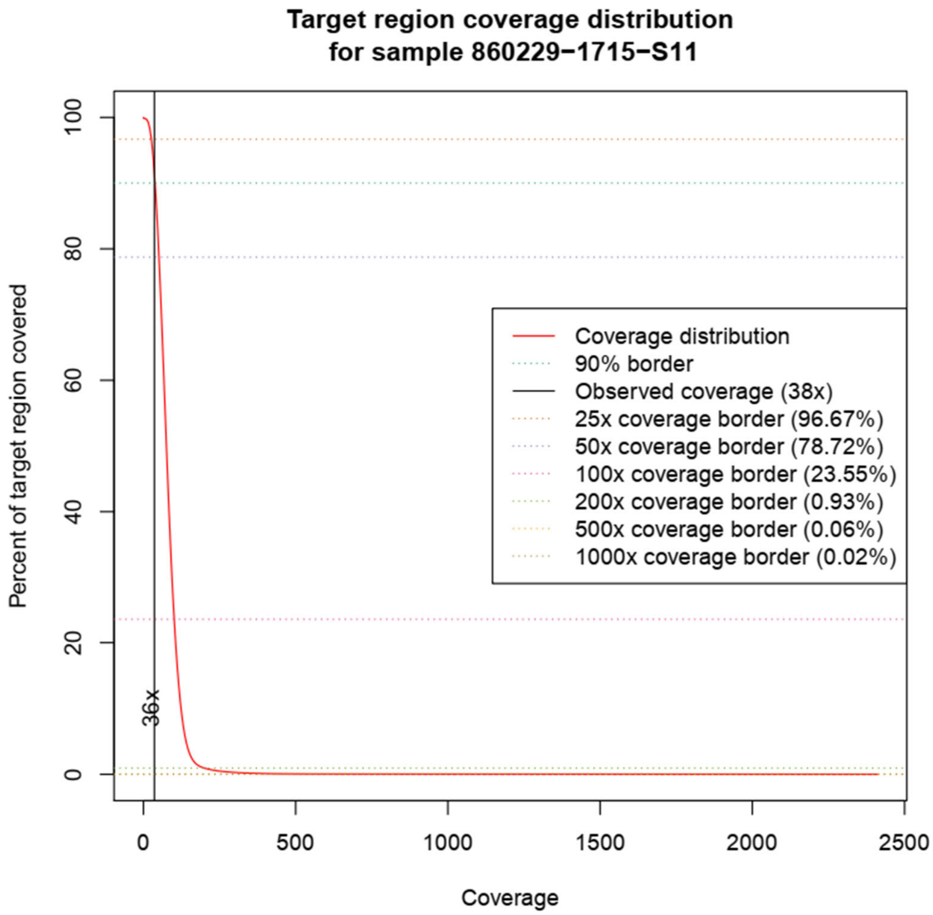

Target region coverage distribution
for sample 860229−1715−S11
Percent of target region covered
Coverage
Coverage distribution
90% border
Observed coverage (38x)
25x coverage border (96.67%)
50x coverage border (78.72%)
100x coverage border (23.55%)
200x coverage border (0.93%)
500x coverage border (0.06%)
1000x coverage border (0.02%)
36x
0
20
40
60
80
100
0
500
1000
1500
2000
2500

II:5

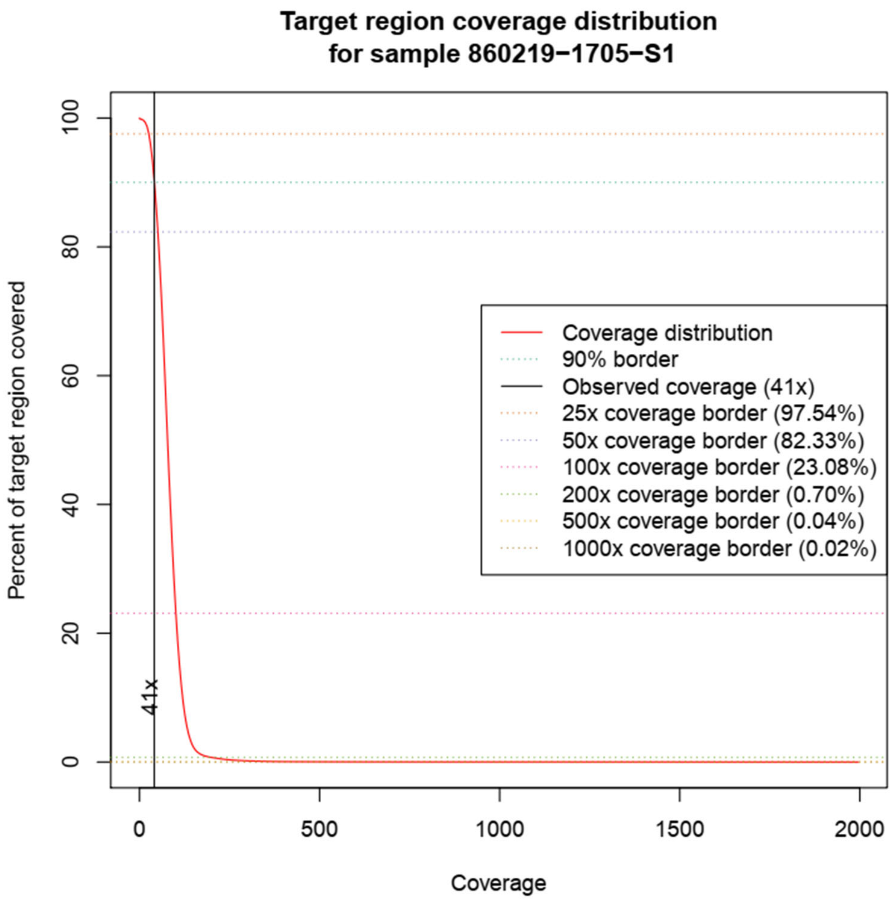

Target region coverage distribution
for sample 860219−1705−S1
Percent of target region covered
Coverage
0
20
40
60
80
100
0
500
1000
1500
2000
41x
Coverage distribution
90% border
Observed coverage (41x)
25x coverage border (97.54%)
50x coverage border (82.33%)
100x coverage border (23.08%)
200x coverage border (0.70%)
500x coverage border (0.04%)
1000x coverage border (0.02%)

III:4

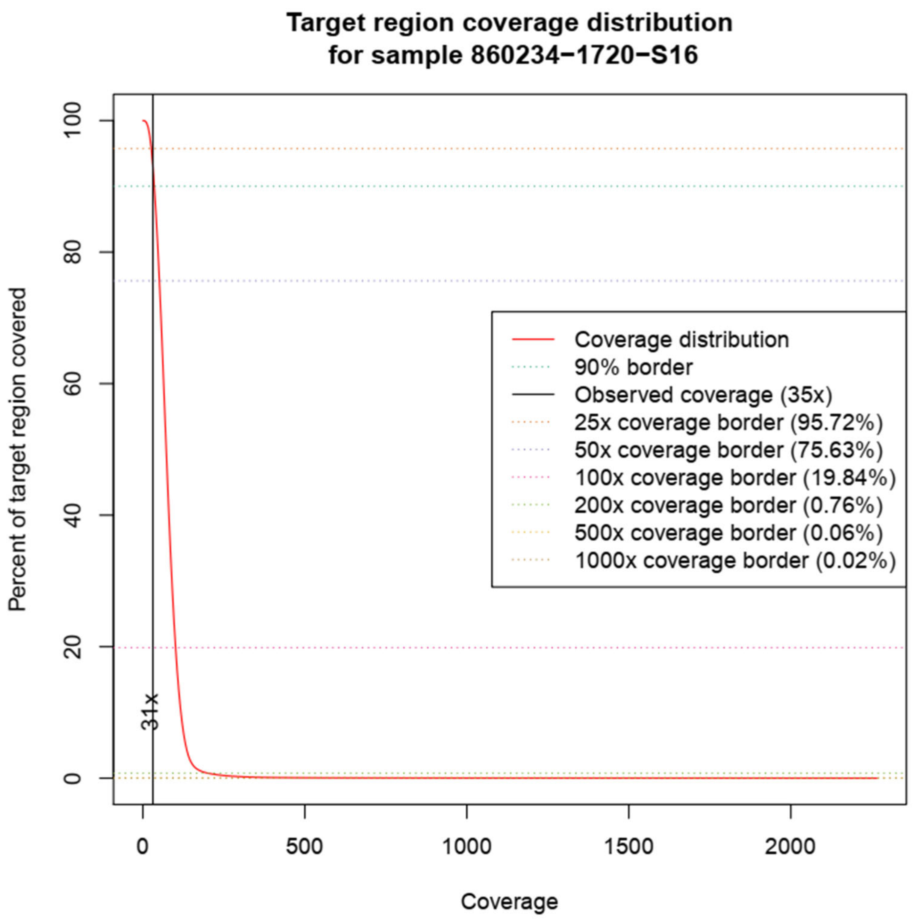
Target region coverage distribution
for sample 860234−1720−S16
Percent of target region covered
Coverage
0
20
40
60
80
100
0
500
1000
1500
2000
31x
Coverage distribution
90% border
Observed coverage (35x)
25x coverage border (95.72%)
50x coverage border (75.63%)
100x coverage border (19.84%)
200x coverage border (0.76%)
500x coverage border (0.06%)
1000x coverage border (0.02%)

III:6

Figure S2. A: Structural analysis of the wild-type FBXO7 protein (AlphaFold AF-Q9Y3I1-F1). B: Interactions of the I74 residue within the FBXO7 protein (DynaMut2)

A

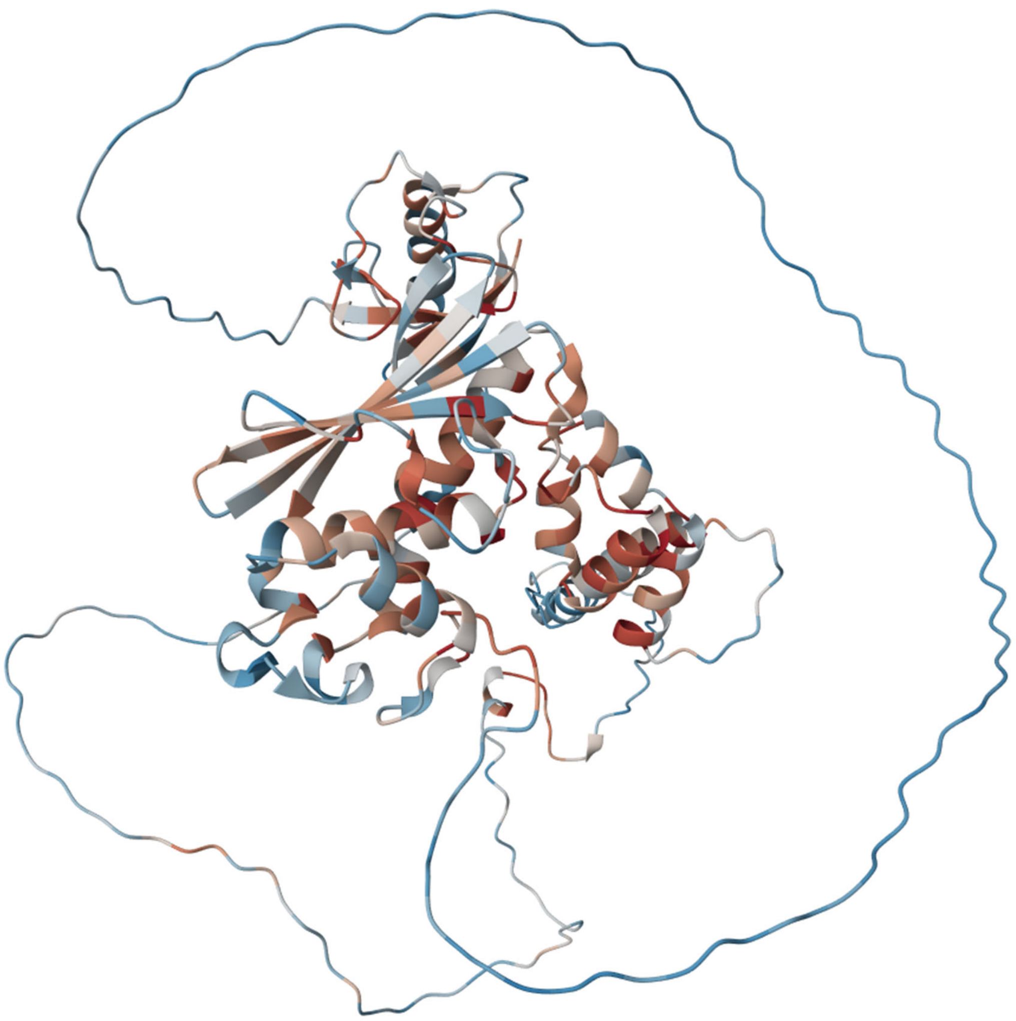

B.

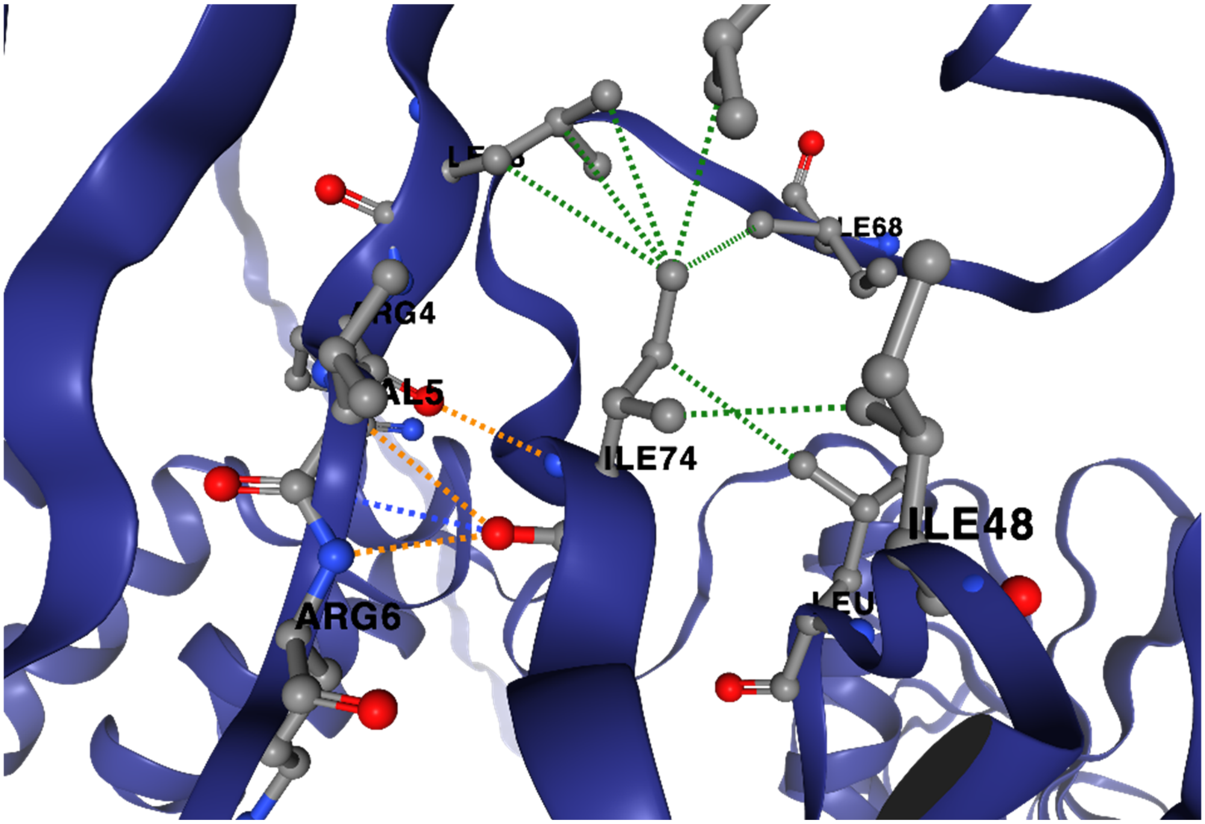

Figure S3. Predicted effect of NM_012179.4:c.[698_703delins] variant on NP_036311.3. A: domain architecture of wild-type and truncated protein. B: Structural models of wild-type and truncated protein

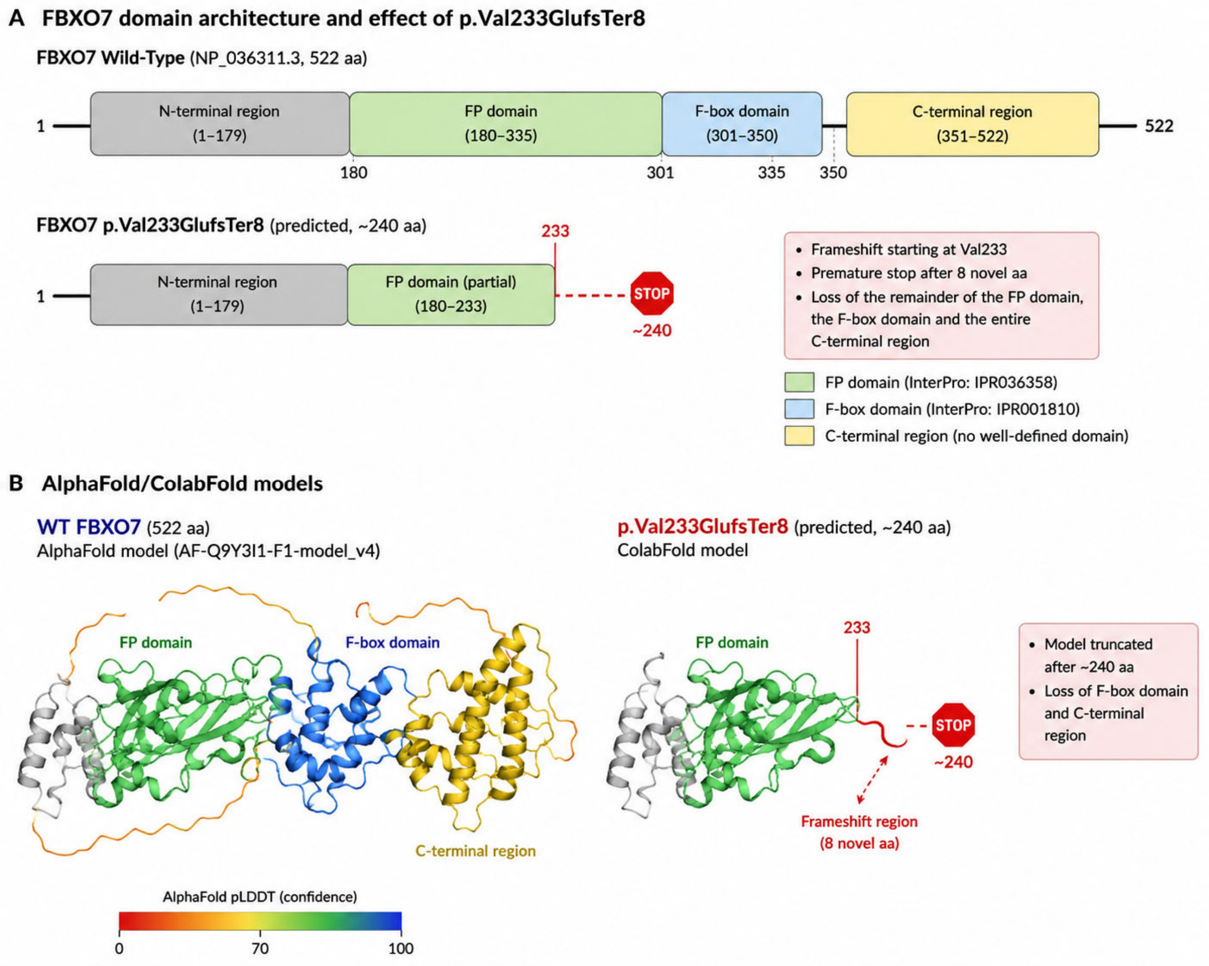

Supplement: Supplementary file 1 [file genes-17-00764-s001.zip › genes-4372834-supplementary.pdf]
